# Alternative Splicing in Oncogenic Kinases: From Physiological Functions to Cancer

**DOI:** 10.1155/2012/639062

**Published:** 2011-10-05

**Authors:** Sabine Druillennec, Coralie Dorard, Alain Eychène

**Affiliations:** ^1^Institut Curie, 91405 Orsay, France; ^2^INSERM U1021, Centre Universitaire, 91405 Orsay, France; ^3^CNRS UMR 3347, Centre Universitaire, 91405 Orsay, France

## Abstract

Among the 518 protein kinases encoded by the human kinome, several of them act as oncoproteins in human cancers. Like other eukaryotic genes, oncogenes encoding protein kinases are frequently subjected to alternative splicing in coding as well as noncoding sequences. In the present paper, we will illustrate how alternative splicing can significantly impact on the physiological functions of oncogenic protein kinases, as demonstrated by mouse genetic model studies. This includes examples of membrane-bound tyrosine kinases receptors (FGFR2, Ret, TrkB, ErbB4, and VEGFR) as well as cytosolic protein kinases (B-Raf). We will further discuss how regular alternative splicing events of these kinases are in some instances implicated in oncogenic processes during tumor progression (FGFR, TrkB, ErbB2, Abl, and AuroraA). Finally, we will present typical examples of aberrant splicing responsible for the deregulation of oncogenic kinases activity in cancers (AuroraB, Jak2, Kit, Met, and Ron).

## 1. Introduction

The process of alternative splicing increases the complexity of the proteome encoded by higher eukaryotic genomes through the synthesis of different products from a single gene. Genes encoding for protein kinases do not escape this rule. While the whole human genome sequencing revealed 518 protein kinases encoding genes [1], a recent bioinformatics survey identified a larger repertoire of about 918 putative protein kinases represented mainly by splice variants from these 518 human genes [2]. This retrospectively gives credit to Tony Hunter's previous witty estimate of “A thousand and one protein kinases” [3]. Protein kinases are among the largest families of genes in eukaryotes, representing approximately 1.7% of all human genes [1]. They are implicated in most of the essential cellular functions, and their deregulation often leads to pathological disorders, including cancer. Alteration of the expression and/or the activity of a number of protein kinases by point mutations, chromosomal translocations, or epigenetic mechanisms can directly initiate or contribute to the development of tumors. The first example came from the seminal discovery of the existence of cellular oncogenes, when the tyrosine kinase c-Src was identified thanks to its retroviral counterpart in the Rous Sarcoma Virus [4]. Since then, numerous oncogenic protein kinases have been discovered, among which EGFR, Abl, Kit, and B-Raf represent typical examples of success in the development of targeted drug therapy [5–7]. While in most cases constitutive activation of the protein catalytic activity is achieved by mutation or overexpression, aberrant splicing has been sometimes incriminated in the deregulation of oncogenic kinases in cancer. In addition, although numerous publications have reported the modulation of kinase activity of oncogenic kinases through alternative splicing, most of these studies have been conducted only *in vitro*. The physiological consequences of alternative splicing in genes encoding oncogenic kinases were not frequently investigated in nonpathological conditions. In the present review we will provide examples of *in vivo* studies exploiting mouse models that clearly disclosed specific physiological functions for naturally occurring splice variants of oncogenic protein kinases. We will also discuss some examples of oncogenic kinases whose normal or aberrant splice variants are associated with a potential role in tumor progression.

## 2. Mechanisms of Oncogenic Kinase Functional Regulation through Alternative Splicing

Alternative splicing allows a single primary transcript to be spliced in several distinct mature mRNAs leading to expression of protein isoforms with different structural and functional properties [8]. One can distinguish two main categories of mechanisms by which alternative splicing can functionally regulate oncogenic protein kinases. The first class includes alternative splicing which maintains the catalytic activity whereas the second class consists of protein products that have lost a functional kinase domain (Table 1).

**Figure 1:**
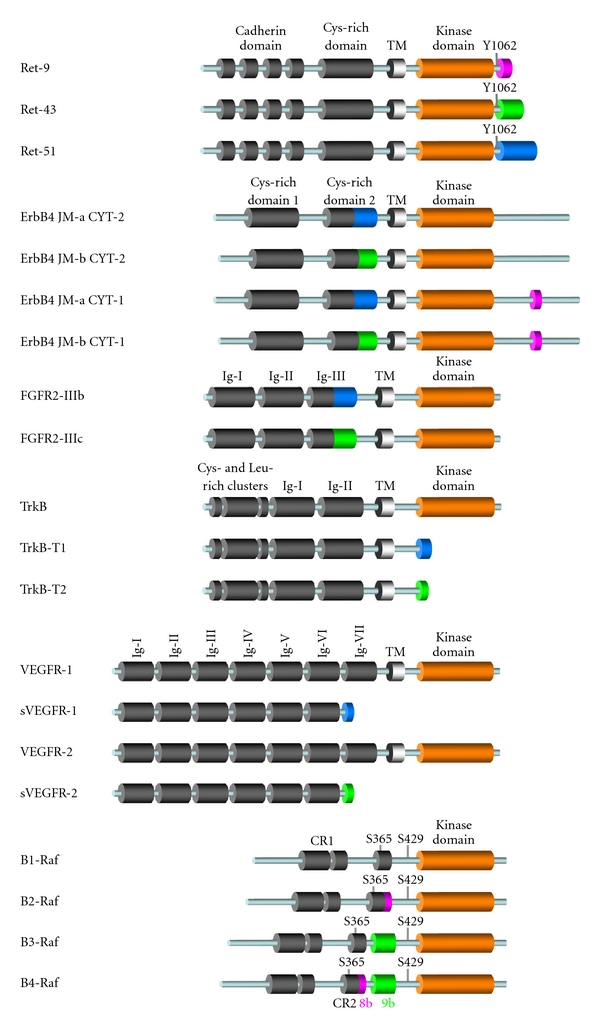
Splice variants of oncogenic kinases studied in mouse models. The various structural domains of oncogenic kinases are represented by cylinders. Amino acid sequences encoded by alternatively spliced exons are represented in pink, green, and blue. Abbreviations: transmembrane domain (TM), immunoglobulin-like domain (Ig), conserved region (CR), tyrosine (Y), and serine (S).

In the first category, the incorporation of alternatively spliced sequences within the open reading frame keeps intact the kinase domain. In most cases for which functional studies have been conducted, such a mechanism allows a fine-tuned regulation of the kinase activity depending on the presence or absence of these additional amino acid sequences. At the molecular level, the incorporated sequences can impact on the catalytic activity by several distinct mechanisms. Posttranslational modifications such as phosphorylation are frequently used to regulate protein kinases activity. For example, autophosphorylation on Tyr-397 of FAK, a nonreceptor tyrosine kinase that acts as a primary regulator of focal adhesion signaling to regulate cell proliferation, survival, and migration, is a critical event, allowing binding of Src family kinases and activation of downstream signaling pathways. Molecular studies have shown that tissue-specific alternative splicing can dramatically alter the mechanism of autophosphorylation of FAK, thereby modifying its oncogenic activity [9]. Likewise, B-Raf isoforms generated by alternative splicing are differentially regulated by phosphorylation mechanisms in a complex manner [10, 11]. B-Raf is a member of the Raf family of cytosolic serine/threonine kinases, which act as Ras effectors and MEK kinases in the canonical RAS/RAF/MEK/ERK signaling pathway [12]. Phosphorylation on both Ser-365 and Ser-429 participates in the differential regulation of B-Raf isoforms through distinct mechanisms (Figure 1). Alternatively spliced exon 8b favors Ser-365 phosphorylation, leading to inhibition of the kinase activity whereas B-Raf isoforms containing alternatively spliced exon 9b are less efficiently phosphorylated on this residue, in agreement with their elevated activity. In contrast, Ser-429 is equally phosphorylated in all B-Raf isoforms, but its phosphorylation differentially regulates the activity of B-Raf, resulting in activation or inhibition of 9b- and 8b-containing isoforms, respectively [11]. 

In several instances, incorporation of additional sequences within the protein imposes novel structural constraints, which can modify intramolecular interactions. Thus, alternative splicing in the linker region of both B-Raf and Fyn, a member of Src family tyrosine kinases, modulates the autoinhibition imposed by the N-terminus regulatory domain on the C-terminus catalytic domain [11, 13].

Another mechanism by which alternative splicing can interfere with the biochemical and biological activity of oncogenic kinases is the change in the repertoire of their partners. This can either be dependent or independent on phosphorylation and the structural constraints mentioned above. A typical example is provided by the gene encoding the tyrosine kinase receptor Ret. Differential splicing of Ret transcripts results in the generation of three isoforms, Ret9, Ret43, and Ret51, whose C-terminal amino acid tails differ after Tyr-1062, and which display differential oncogenic activities (Figure 1) [14]. Amino acid changes in the sequences surrounding Tyr-1062 residue are responsible for the differential binding on phosphorylated Tyr-1062 of several key signaling molecules such as Shc, Grb2, and the ubiquitin ligase Cbl [15–17]. 

The gene encoding ErbB4, another tyrosine kinase receptor, also undergoes complex alternative splicing producing both juxtamembrane (JM-a and JM-b) and cytoplasmic (CYT-1 and CYT-2) isoforms (Figure 1). The 16-amino acid CYT-1 specific sequence, which is absent from CYT-2 isoforms, contains a binding site for the p85 regulatory subunit of PI3K [18]. In addition, it mediates ErbB4 ubiquitination and endocytosis by providing a binding site for the WW domain-containing HECT-type E3 ubiquitin ligase Itch. Consequently, CYT-1 isoforms are efficiently endocytosed whereas CYT-2 isoforms are impaired in endocytosis [19]. The JM-a isoforms contain a metalloprotease cleavable extracellular domain whereas JM-b isoforms are resistant to cleavage [20]. The ligand-dependent proteolysis of ErbB4 JM-a isoforms releases soluble intracellular domains of 80 kDa, s80Cyt2, and s80Cyt1, which exhibit nuclear cytoplasmic shuttling and differ by the above mentioned 16 amino acid CYT-1 specific sequence absent from CYT-2 isoforms [18, 19].

The fibroblast growth factor (FGF) receptor (FGFR) family of tyrosine kinases provides another remarkable example of how alternative splicing can be used to vary the repertoire of functional interactions with profound physiological consequences. The FGF family of ligands comprises 22 members, and FGFR signaling activity is regulated by the binding specificity of ligands and receptors [21]. This specificity largely relies on the immunoglobulin-like loop III of the extracellular ligand-binding domain of FGFRs [21]. Three of the four FGFRs, FGFR1, 2, and 3, express splice variants, which alternatively use exon 8 or 9 to encode the C-terminal half of this immunoglobulin-like domain III, generating IIIb and IIIc receptor isoforms with different affinities for FGFs (Figure 1) [22]. For instance, FGFR2-IIIb binds FGF7 and FGF10, but not FGF2 whereas the FGFR2-IIIc isoform binds FGF2 and FGF18, but not FGF7 and FGF10 [21]. This provides each isoform a specific physiological function during development, which is further described in the next section.

The second main class of oncogenic kinase splice variants is composed of truncated proteins devoid of the catalytic kinase domain (Table 1). Such alternative splicing often results in the synthesis of a polypeptide endowed with dominant negative properties. However, this does not appear to be always the case and putative specific and kinase-independent functions have been proposed for such splice variants. A well-characterized dominant negative function was reported in the case of A-Raf, a cytosolic kinase closely related to B-Raf [12]. The A-Raf gene undergoes alternative splicing, which generates truncated isoforms containing the Ras-binding domain but lacking the kinase domain. These splicing isoforms, whose expression is tightly regulated by extra and intracellular cues, prevent ERK signaling activation by titrating the upstream activators Ras GTPases [23, 24]. However the physiological relevance of these observations await *in vivo* evidence.

The generation of truncated isoforms lacking a kinase domain is frequently observed within the family of membrane-bound tyrosine kinase receptors. Trk receptors are a family of three tyrosine kinases (TrkA, TrkB, and TrkC) activated by neuropeptides of the neurotrophin family, thereby regulating cell survival, proliferation, and the fate of neural precursors [25]. In addition to their roles in the adult nervous system, where they regulate synaptic strength and plasticity, Trk receptor deregulation has been also implicated in human cancers. Complex alternative splicing results in the expression of different truncated TrkB and TrkC receptors lacking the kinase domain, whose functions are not completely understood [25, 26]. Despite some evidence suggesting that truncated receptors can trigger intracellular signaling alone, tyrosine kinase activity appears to be necessary for most Trk receptor-mediated responses to neurotrophins. It has been proposed that the truncated receptors can raise the local effective neurotrophin concentration by sequestering and/or presenting neurotrophins to neurons expressing full-length Trk receptors. However, when expressed on the same neuron as a full-length Trk receptor, truncated receptors inhibit ligand-induced activation of Trk kinase activity by forming nonproductive heterodimers, thereby acting as dominant negative proteins [25]. For example, the human TrkB gene encodes C-terminal truncated receptors; TrkB-T1 and TrkB-T2, which are generated by the alternative usage of exon 16 or exon 19, respectively, containing translational stop codons (Figure 1) [25, 26]. TrkB is the functional receptor for brain-derived neurotrophic factor (BDNF) and both TrkB-T1 and TrkB-T2 have the ability to bind it. Although they can act as dominant negative inhibitors of the full-length receptor by preventing ligand-induced phosphorylation, it has been suggested that the truncated isoforms may have signaling properties distinct from those of the full-length TrkB, such as evoking calcium signaling and mediating Rho GDP dissociation inhibitor 1 function [27–29].

Another example of kinase domain truncation by alternative splicing is provided by the VEGFR (vascular endothelial growth factor (VEGF) Receptor) family, which encodes membrane-bound tyrosine kinase receptors for VEGFs acting as key regulators of vasculogenesis, angiogenesis, and lymphangiogenesis, both in physiological conditions and in cancer [30, 31]. VEGF-A binds to both VEGF receptor 1 (VEGFR-1, also named Flt-1) and VEGFR-2 (also named KDR), thereby regulating vasculogenesis and angiogenesis respectively whereas VEGF-B binds only to VEGFR-1 regulating vasculogenesis. On the other hand, VEGFR-2 is also involved in lymphangiogenesis through activation by VEGF-C and VEGF-D. VEGFR-1 and VEGFR-2 undergo alternative splicing of their cytoplasmic C-terminal domain resulting in truncated receptors (Figure 1) [32–34]. However, unlike the Trk family members, both the kinase and transmembrane hydrophobic domains are eliminated, thereby generating secreted soluble extracellular domains that have retained their ligand-binding capacity and act as ligand traps.

The following section will review in depth examples of the physiological consequences of the various mechanisms described above. In addition, other mechanisms of regulation by alternative splicing were reported in very specific cases of tumor-associated splice variants of protein kinases. These will be discussed in the last section.

## 3. Physiological Functions of Oncogenic Kinase Splice Variants Revealed by Genetically Engineered Mouse Models

As described above, alternative splicing can be used either to allow subtle regulation of protein kinase activity or to provide additional or dominant negative functions to these proteins, suggesting that the impact of alternative splicing on physiological processes can strongly vary depending on the mechanism. Although alternative splicing has been reported in a vast number of oncogenic kinases encoding genes, experimental evidence for a physiological role is lacking for most of the cases. In this section we will describe typical examples of specific functions attributed to splicing variants of oncogenic kinases revealed by genetically engineered mouse models. These *in vivo* studies have highlighted the physiological importance of alternative splicing in both fundamental processes during development and more specialized functions such as behavior.

### 3.1. FGFR2

As mentioned above, alternative splicing in the extracellular domain of FGFRs results in differential binding of various FGFs. In general, the IIIb and IIIc isoforms of FGFRs are often expressed on epithelial and mesenchymal cells, respectively, while their specific ligands show an opposite pattern of expression, allowing signaling to occur in a paracrine fashion between adjacent tissues [21, 22]. Among FGFRs, alternative splicing of FGFR2 receptor has been the best studied in animal models (Figure 1). FGFR2-IIIb is expressed in the surface ectoderm and in the endothelium of internal organs throughout development, while FGFR2-IIIc is expressed in the paraxial and lateral mesoderm, in the limb bud and branchial arch mesenchyme and later in muscle and other mesenchymal tissues, including the periphery of bone forming cartilage models [35, 36]. The specific functions of both FGFR2-IIIb and FGFR2-IIIc isoforms were investigated in mouse models using different strategies. With regard to FGFR2-IIIb, two different mouse lines deficient for FGFR2-IIIb were generated, either by classical exon 8 ablation or by introducing a stop codon and an IRES-LacZ transgene in exon 8 [35, 37]. Noteworthily, the expression of FGFR2-IIIc, the other alternatively spliced isoform, was not affected, and both models resulted in similar phenotypes. The loss of FGFR2-IIIb abrogates limb outgrowth with multiple defects in branching morphogenesis, a phenotype similar to that of the loss of function mutation of FGF10, which binds FGFR2-IIIb but not FGFR2-IIIc. Although viable until birth, FGFR2-IIIb null mice display severe defects of the limbs, lung, and anterior pituitary gland [35, 37]. The development of these structures appears to initiate, but then fails with the tissues undergoing extensive apoptosis. FGFR2-IIIb null mice also show developmental abnormalities of the salivary glands, inner ear, teeth, and skin, as well as minor defects in skull formation [35]. 

The specific functions of the other receptor isoform, FGFR2-IIIc, were also investigated using either a knockout approach eliminating exon 9 or a knockin strategy introducing a stop codon in exon 9 [36, 38]. In contrast with FGFR2-IIIb studies, the results from both strategies used for FGFR2-IIIc were not fully concordant. Knockin-mediated loss of FGFR2-IIIc expression resulted in a recessive viable phenotype with craniosynostosis and retarded development of the axial and appendicular skeleton, causing dwarfism and misshapen skull [36]. In exon 9 knockout mice, however, heterozygotic ablation of FGFR2-IIIc isoform resulted in a gain-of-function mutation, characterized by neonatal growth retardation and death, coronal synostosis, ocular proptosis, precocious sternal fusion, and abnormalities in secondary branching in several organs that undergo branching morphogenesis [38]. The discrepancies observed between the two models could be explained by the fact that the knockin mutation causes a splice switch, inducing ectopic expression of the FGFR2-IIIb isoform. These results highlight the differences that can arise from different *in vivo* approaches and underline the significance of careful experimental design.

### 3.2. Ret

Mouse models have been generated to investigate the respective physiological functions of Ret9 and Ret51 (Figure 1), the two major isoforms of Ret, a receptor previously reported to play a critical role in kidney organogenesis and the development of the enteric nervous system [39]. Partial cDNAs encoding the C-terminal intracellular part of human Ret9 or Ret51 were inserted in frame into exon 11 of the mouse locus, immediately after the segment encoding the transmembrane domain, thereby generating mice that express either Ret9 or Ret51 isoforms. Ret9 mice, which lack Ret51, were viable and appeared normal, demonstrating that the expression of Ret9 alone is sufficient to support normal embryogenesis and postnatal life. In contrast, Ret51 mice, which lack Ret9, displayed severe innervation defects resulting in kidney hypodysplasia and a lack of enteric ganglia from the colon [39]. As mentioned above, both Ret isoforms differ by the sequence context of the multidocking phosphorylated Tyr-1062 site. The generation of knockin mice in which Tyr-1062 of either Ret9 or Ret51 was mutated to phenylalanine demonstrated that this residue plays a critical role in specific Ret9 requirement in the development of the kidneys and the enteric nervous system [40]. Interestingly, these findings provide direct *in vivo* evidence that the distinct activities of Ret9 and Ret51 isoforms result from the differential regulation of Tyr-1062-mediated signaling through alternative splicing of C-terminal flanking sequences.

### 3.3. ErbB4

The case of ErbB4 appears more complex because of alternative splicing both in the extracellular juxtamembrane region and in the cytoplasmic C-terminal tail, giving rise to four distinct isoforms with differential tissue-specific expression (Figure 1). ErbB4, also known as HER4, belongs to the ErbB/HER/EGFR family of tyrosine kinase receptors. It binds to and is activated by neuregulins-2 and -3, heparin-binding EGF-like growth factor and betacellulin [41]. As mentioned above, the ligand-induced proteolysis of ErbB4 JM-a isoforms releases soluble intracellular domains of 80kDa called s80Cyt2 and s80Cyt1, which exhibit nuclear cytoplasmic shuttling and differ by the 16-amino acid CYT-1 specific sequence absent from CYT-2 isoforms [18, 19]. Owing to the lack of specific knockout mouse models, the respective contribution of the different ErbB4 isoforms to neuregulin-induced signaling remains poorly understood. However, Muraoka-Cook et al. generated transgenic mice to compare the effect of induced expression of s80Cyt1 or s80Cyt2 on mammary epithelium *in vivo* [42]. These mice express the GFP-tagged intracellular domain of either ErbB4-Cyt1 or ErbB4-Cyt2 isoforms under the control of a doxycycline-inducible reverse tetracycline transcriptional activator- (rtTA-) dependent promoter. Induced expression of either isoform in the mammary gland was obtained by crossing with transgenic mice expressing rtTA under the control of the mouse mammary tumor virus LTR. Interestingly, the resulting phenotypes revealed that the s80Cyt1 and s80Cyt2 isoforms exert markedly opposing effects on mammary epithelium growth and differentiation. Expression of s80Cyt1 during puberty decreased proliferation and caused precocious lactogenesis in the ductal epithelium of virgin mice. In contrast, expression of s80Cyt2 caused epithelial hyperplasia, characterized by increased Wnt and nuclear *β*-catenin expression, and elevated expression of c-myc and cyclin D1 in the mammary epithelium. Although these models do not allow assigning specific physiological functions to the four ErbB4 isoforms, they clearly indicate that the single insertion of 16 amino acids in the C-terminal part of the receptor via alternative splicing is sufficient to confer ErbB4 isoforms opposite biological effects.

### 3.4. VEGFR-1 and VEGFR-2

VEGFR-1 and VEGFR-2 arose through gene duplication during evolution resulting in a highly similar exon-intron structure. Consequently, both genes are subjected to the same alternative splicing mechanism giving rise to the production of secreted soluble extracellular domains, named sVEGFR-1 and sVEGFR-2 (Figure 1), that have retained their ligand-binding capacity [32, 33]. Since their discovery, a putative ligand trap role has been proposed for such splice variants, which was further exploited for the development of VEGF-targeted antitumor therapies [30]. However, the direct demonstration of physiological functions for both sVEGFR-1 and sVEGFR-2 soluble isoforms has come from studies in mice.

The first mouse model demonstrated that sVEGFR-1 can act as a positive modulator of vascular sprout formation and branching morphogenesis [43]. In the course of their investigation on the effects of a VEGFR-1^−/−^ null mutation on blood vessel formation, the authors noticed aberrant morphogenesis of embryonic vessels. This phenotype was rescued with a sVEGFR-1 transgene suggesting a model whereby sVEGFR-1 isoform locally traps VEGF-A to establish or modify a gradient that regulates vascular sprouting and endothelial cell migration. The role of sVEGFR-1 was also investigated in cornea avascularity using three complementary strategies in the mouse [44]. Corneal avascularity is required for optical clarity and optimal vision. The molecular mechanisms responsible for this absence of blood vessels in the cornea have remained enigmatic for a long time, owing to the presence of VEGF-A in the cornea and its proximity to vascularized tissues. Using neutralizing antibodies, RNA interference and Cre-lox-mediated gene disruption, Ambati and colleagues have demonstrated that the presence of sVEGFR-1 is necessary and sufficient to trap VEGF-A in the cornea, thereby inhibiting angiogenesis [44]. Interestingly, the mouse cornea also expresses sVEGFR-2, but not the full-length VEGFR-2 receptor [33]. Loss of sVEGFR-2 expression in the cornea obtained by tissue-specific targeted disruption of the VEGFR-2 gene resulted in lymphatic invasion of the normally alymphatic cornea at birth. These results show that sVEGFR-2 act as an endogenous inhibitor of lymphangiogenesis by trapping VEGF-C.

### 3.5. TrkB

The putative physiological role of the alternatively spliced TrkB-T1 truncated receptor has been initially investigated in transgenic mice over expressing it in postnatal cortical and hippocampal neurons [45, 46]. These animals have impaired long-term spatial memory, a phenotype similar to that of BDNF knockout mice [47], and show increased susceptibility to cortical injury after focal cerebral ischemia. Therefore, these studies suggested that TrkB-T1 could indeed act as a dominant negative receptor inhibiting BDNF signaling, at least in some types of brain functions. More recently, however, the functions of TrkB-T1 were reassessed in a knockout mouse model with a selective ablation of this isoform that did not affect the spatiotemporal expression of the full-length receptor [48, 49]. Surprisingly, TrkB-T1-deficient mice do not show any overt phenotype, although they are more anxious than their control littermates and display morphological changes in the length and complexity of neurites of the basolateral amygdala neurons. Moreover, although loss of TrkB-T1 does not affect normal brain development or function, its reduction can improve deficiencies associated with BDNF haploinsufficiency, thereby clearly demonstrating that TrkB-T1 can limit BDNF/TrkB signaling in a physiological manner. 

### 3.6. B-Raf

The studies described above have provided various examples of the physiological relevance for alternative splicing of genes encoding membrane-bound tyrosine kinase receptors otherwise implicated in human cancers. In contrast, only one study has been conducted *in vivo* to investigate such physiological functions of splice variants of a cytosolic oncogenic protein kinase, thus far. As already mentioned, the B-*raf* gene encodes multiple isoforms displaying tissue-specific expression [50, 51]. The kinase activity of the different B-Raf isoforms is modulated by the presence or absence of sequences encoded by exon 8b and 9b, located in the hinge region upstream of the kinase domain (Figure 1). While exon 9b is conserved in vertebrates, including fish, amphibians, avians, and mammals, exon 8b appeared later during evolution since it is found only in eutherians, but not in other mammals such as marsupialia and monotremata [52]. To investigate the physiological relevance of B-*raf* alternative splicing during development, conditional knockout mice for either exon 8b or exon 9b were generated. In contrast with the complete B-*raf* knockout, none of these exons were required for normal development, since adult animals carrying a homozygous constitutive mutation (B-*raf*Δ8b/Δ8b or B-*raf*Δ9b/Δ9b) survived up to at least 18 months in the absence of detectable anomalies [52]. However, behavioral analyses revealed that expression of exon 9b-containing isoforms was required for B-Raf function in hippocampal-dependent learning and memory. In contrast, mice carrying a mutation of exon 8b were not impaired in this function. It has been proposed that the requirement for exon 9b-containing isoforms was due to their resistance to PKA-mediated inhibitory phosphorylation on Ser-365 and Ser-429 (Figure 1), allowing convergence of PKA and ERK signaling on nuclear targets involved in the process. Interestingly, this study also revealed that the putative function of exon 8b-containing B-Raf isoforms, which remains to be characterized, has probably evolved during evolution since alternative splicing of exon 8b appears differentially regulated in primates, as compared with other mammals [52].

## 4. Alternative Splicing of Oncogenic Kinases and Cancer

As for most oncogenes and tumor suppressor genes, perturbations in alternative splicing can modify the expression and activity of oncogenic kinases during tumor development and maintenance. Such phenomenons sometime proceed from the deregulation of an otherwise normal physiological alternative splicing, as illustrated below by examples from the FGFR, ErbB, and Trk families of tyrosine kinase receptors. In other cases such as Abl or Aurora kinases, it is not clearly established whether the alternative splicing mechanism implicated in tumor progression also plays physiological roles in the nonpathological functions of the kinase. Finally, Jak2, Kit, Ron, and Met kinases provide typical examples of aberrant splicing generating oncogenic splice variants that are not usually observed in nonpathological conditions. We will review here some of these, by focusing specifically on oncogenic kinases whose splicing profiles are associated with an established functional consequence in cancer. 

### 4.1. FGFRs

The involvement of FGFR2 spliced isoforms in developmental processes was described above. Interestingly, alternative splicing of FGFR2 also appears to be implicated in cancer. FGFR2 exon switching from the IIIb to the IIIc isoform has been observed during epithelial cell tumor progression, notably in breast cancer [53–55]. FGFR2 IIIc expression is associated with a loss of epithelial markers and a gain of mesenchymal markers. Furthermore, the C-terminus of FGFR2-IIIb also undergoes additional alternative splicing, thereby modulating its oncogenic activity [54–56]. Thus, breast cancer progression may involve two distinct alternative splicing events, first involving the expression of FGFR2 IIIb C2/C3 isoforms associated with progression from normal breast epithelium to noninvasive breast cancer, and secondly involving the conversion to the mesenchymal FGFR2 IIIc isoform in invasive breast cancer cells [55].

Studies in cancer cells further revealed an even more complicated regulation of the FGFR receptors family activity by alternative splicing [57]. For example, FGFR1 exhibits another RNA splicing event that results in skipping of the exon encoding the Ig-like domain I [58]. The receptor containing only two Ig-like domains (FGFR1-*β*) displays a higher binding affinity for FGF1 [59, 60] and, unlike the full-length FRFR1-*α* isoform, caused cancer in a model of nude mice xenograft [61]. An increase in the FGFR1-*β* levels has been associated with tumor progression, reduced relapse-free survival, and malignancy in astrocytomas, breast, pancreatic, and bladder cancers [62–64]. However, little is known about the biological and functional consequences of increased expression of FGFR1-*β* in cancer cells.

### 4.2. ErbB2

The transmembrane HER2/ErbB2/Neu tyrosine kinase receptor belongs to the epidermal growth factor receptor (EGFR) family. Overexpression and gene amplification of ErbB2 are frequently observed in human malignancies, in particular in 25–30% of primary human breast cancers [65]. This correlates with enhanced tumor aggressiveness and poor patient outcome. Several studies have reported the expression of an ErbB2 alternatively spliced isoform in normal mammary cells and in human breast carcinomas [66]. The in-frame deletion of 16 amino acids in the juxtamembrane domain due to exon 16 splicing induces the formation of ΔErbB2 that displays a stronger transforming activity than wildtype ErbB2. Structural analyses of the ErbB2 extracellular domain have shown that electrostatic repulsions may probably prevent homodimerization of this receptor [67]. Splicing in ΔErbB2 is supposed to trigger the kinase activity by promoting intermolecular disulfide bonding and, in turn, homodimers capable of transforming cells. Levels of the ErbB2 splice variant are only 5% of those observed with the wildtype receptor, both in primary normal breast tissue and breast cancers. ErbB2 gene amplification in primary human breast cancer might increase the levels of this oncogenic variant above a critical threshold, therefore allowing it to contribute to breast cancer progression [68]. 

### 4.3. TrkB

As previously mentioned, studies using knockout mice demonstrated that at physiological levels, TrkB-T1 acts as a dominant negative receptor inhibiting BDNF signaling in brain neurons. However, *in vitro* studies suggest that TrkB-T1 may also have signaling properties on its own [27–29]. In support of this, overexpression of TrkB-T1 but not a TrkB-T1 COOH-terminal deletion mutant in nonmetastatic pancreatic cancer cells was shown to induce liver metastasis in an orthotopic xenograft mouse model of pancreatic cancer by sequestering Rho GDP dissociation inhibitor and promoting RhoA activation [69]. Accordingly, TrkB-T1 but not full-length TrkB is overexpressed in pancreatic cancer cell lines and pancreatic tumor samples [69].

### 4.4. Aurora

Aurora kinases are a family of serine and threonine protein kinases that function as key regulators of mitosis by controlling the accurate and equal segregation of chromosomes. Two members, Aurora-A and -B, are frequently overexpressed in a wide variety of human cancers. Aberrant expression of these kinases correlates with poor prognosis and is implicated in oncogenic transformation mainly by inducing chromosomal instability. The expression of different 5′UTR alternative splicing variants of Aurora-A was first reported in breast cancer cell lines and primary tumors [70]. Lai et al. also described that the exon 2-containing variant, which contributes to Aurora-A overexpression under EGF treatment, is dominantly expressed in colorectal cancer tissues compared to normal human colon tissues [71]. The recruitment of specific splicing variants of Aurora-A mRNA in regulating Aurora-A protein expression indicates that there could be a potential correlation between exon 2-containing variant and colorectal cancer development. Two Aurora-B alternative splicing variants were also identified in liver cancer cells and tumor biopsies [72]. AURKB-Sv1 retained 46 bp of intron 5 and lost 96 bp of exon 5, while exon 6 is missing in AURKB-Sv2. The variant form was absent in normal liver and was overexpressed in metastatic liver cancer compared to hepatocellular carcinoma (HCC). This aberrant expression was associated with the advanced stages of HCC, and correlated with a poor outcome and a shorter disease-free period. This suggested that AURKB-Sv2 could be a marker of poor prognosis in hepatocarcinogenesis. Further experiments are necessary to elucidate the structural and functional properties of this alternative splicing in HCC. Of note, the putative physiological functions of splice variants of Aurora kinases have not been assessed in mouse models, thus far.

### 4.5. ABL

Myeloproliferative neoplasias (MPNs) are hematopoietic stem cell disorders linked to uncontrolled overproduction of mature and functional blood cells. Chronic myeloid leukemia (CML), the most common MPN, is characterized by a chromosomal translocation, which leads to the production of the breakpoint cluster region-abelson (BCR-ABL) fusion oncoprotein. BCR-ABL contains a constitutively activated tyrosine kinase domain that plays a role in malignant transformation and triggers CML. Numerous BCR-ABL alternative spliced variants have been reported [73–76]. For instance, the 35-bp insertion of ABL intron 8 between exon 8 and exon 9 results in a reading-frame shift and leads to the expression of a truncated protein missing the last 14 residues of the kinase domain and the following C-terminus residues. However, ABL alternative splicing appears to be quite frequent in the normal population and an increase in expression levels and the frequency of these alternatively spliced BCR-ABL associated with imatinib resistance in the chronic phase of CML is still a matter of debate [77–79]. 

### 4.6. JAK2

Non-CML MPNs such as polycythemia vera (PV), essential thrombocythemia (ET), or idiopathic myelofibrosis (IMF) display recurrent anomalies in the JAK2 tyrosine kinase that plays a critical role in mediating hematopoietic cytokine receptor signaling through the JAK/STAT pathway [80]. The JAK2 V617F mutation in exon 14 is the most common single-point mutation found mainly implicated in chronic myeloproliferative disorders (found in 35% to 95% of PV, ET, and IMF patients) [81, 82]. *In vitro*, the V617F substitution increases JAK2 kinase activity, thus causing cytokine-independent growth of cell lines and cultured bone marrow cells. From predicted JAK2 structure analysis, this mutation is believed to disrupt the autoinhibitory interaction between the pseudokinase (JH2) and kinase (JH1) domains thus allowing the activation loop of JH1 to adopt a conformation in which it can be phosphorylated by an adjacent JAK2V617F molecule. Further investigations revealed that around 15% of patients suffering from MPN also express a JAK2 exon 14 mRNA splice transcript accounting for around 12% of total JAK2 transcripts [83, 84]. The deletion of exon 14 (88 bp) leads to the frame shift of seven amino acids followed by a stop codon. The truncation of the JAK2 protein within the pseudokinase domain thus results in deletion within the JH2 domain and complete deletion of the kinase domain (JH1). How this alternative short form of JAK2 is able to activate the JAK2-STAT pathway remains unclear. Additional functional studies are needed to demonstrate the role of the Δexon14 deletion in deregulating JAK2 activity and in leukemogenesis.

### 4.7. Kit

The tyrosine kinase receptor c-Kit and its ligand SCF (stem cell factor) play essential functions in a number of cell types, including hematopoietic stem cells, mast cells, melanocytes, and germ cells. Activating mutations of c-Kit have been described in various human cancers, including melanomas, testicular germinomas, acute myeloid leukemia, and gastrointestinal stromal tumors (GIST). Many different alternative splicing sites in c-Kit were first reported in leukemia cells [85, 86] and then in GIST [87, 88]. In GIST, the loss of 27 nucleotides in exon 11 of c-Kit results in polypeptides that remain in-frame but lack a nine amino acids internal stretch that is crucial for autoinhibition of the kinase. Structural studies of the mutated kinase revealed a conformation consistent with constitutive activation [88].

### 4.8. Met

Signaling mediated by HGF/c-Met promotes a wide range of biological activities including cell growth, morphogenic differentiation, motility, invasion, and angiogenesis. Dysregulation of this pathway has also been implicated in the development and progression of various malignant tumors. Identification of activating germline mutations of c-Met in hereditary papillary renal carcinomas provided the first evidence linking c-Met to human oncogenesis [89]. Afterward, many c-Met missense mutations were observed in a wide variety of cancers. A majority of them are located in the activation loop of the kinase domain. However, somatic intronic mutations leading to an exon 14 in-frame deletion originally observed in fetal mouse tissues, [90] were also reported in approximately 3% of nonsmall cell lung cancers [91–93] and in gastric cancer cell lines [94]. This missense mutation leads to an alternatively spliced transcript that encodes a 47-amino acid deletion in the cytoplasmic juxtamembrane domain thus removing the Y1003 phosphorylation site necessary for Cbl E3-ligase binding. Cellular analyses have shown that the Met deletion mutant receptor displays decreased Cbl binding and ubiquitination, prolonged protein stability, and sustained signaling on ligand stimulation in agreement with enhanced transforming properties [92, 95]. These results are consistent with an important role of the c-Met alternatively spliced form in the development and progression of human cancer.

### 4.9. Ron

Ron, the tyrosine kinase receptor for the Macrophage-stimulating protein belongs to the MET proto-oncogene family. Ron is a heterodimer (p185RON) derived from the proteolytic cleavage of a single-chain common precursor and is composed of a disulfide-linked 40 kDa *α*-chain and a 145 kDa *β*-chain with intrinsic tyrosine kinase activity. With the involvement in cell dissociation, motility, and matrix invasion, Ron has been found to be essential in embryonic development and in tumor invasive/malignant phenotypes. Thus, elevated Ron expression has been found in breast, colon, lung, bladder, and ovarian cancers. Importantly, Ron is expressed as a single sized mRNA resulting from constitutive splicing in normal tissues whereas in cancer cells, the altered expression of Ron is often accompanied by the generation of Ron variants mainly through mRNA splicing [96]. This suggests that a switch from the constitutive to alternative splicing occurs in cancer cells.

A Ron isoform, named RonΔ165, was identified in human gastric carcinoma cells and induces an invasive phenotype in transfected cells [97]. RonΔ165 (165 kDa) is generated by the alternative splicing of exon 11 (147 bp), which leads to the in-frame deletion of a 49-amino acid region in the *β* chain extracellular domain. By inducing a drastic change in the three-dimensional structure through aberrant intermolecular disulfide bridge formation, this deletion enables the proteolytic cleavage thus enabling cellular membrane anchorage of the receptor and rendering the kinase constitutively active in the absence of its ligand in the cytoplasm. Interestingly, abnormal accumulation of RonΔ165 transcripts occurs in primary human colorectal and breast carcinomas and correlates with the metastatic phenotype [98, 99]. Ghigna et al. identified a silencer and an enhancer of splicing within exon 12 that regulates the level of inclusion of exon 11. An increase in RonΔ165 levels is stimulated by binding of the SR protein SF2/ASF to one of these regulatory elements. The authors speculate that SF2/ASF could regulate malignant transformation of certain epithelial tumors by inducing RonΔ165-dependent EMT and that pharmacological treatment of aberrant splicing could lead to new anticancer therapeutic approaches [100].

Two others spliced variants RonΔ155 and RonΔ160 were also identified in human colon carcinoma cell lines and primary tumors [98, 101]. RonΔ160 is derived from a splicing mRNA transcript with an in-frame deletion of 109 amino acids coded by exons 5 and 6 and located in the Ron *β*-chain extracellular sequences. The deletion does not affect the proteolytic processing, but induces structural changes that could stabilize an active form of the membrane receptor by disulfide bridges triggered oligomerization. Therefore, the constitutive activation of RonΔ160 by autophosphorylation increases its cell migration and invasion properties [102]. RonΔ155 presents a combined deletion of exons 5, 6, and 11 in the extracellular domain of the *β*-chain thus preventing its maturation into the *α*/*β* two-chain form. The receptor is constitutively active and capable of inducing tumor formation *in vivo* [98].

## 5. Conclusions and Perspectives

Splice variants have been described in many cancer-associated proteins, and in particular in oncogenic kinases. The biological activity of the majority of alternatively spliced isoforms and their contribution to cancer biology is yet to be elucidated. Nevertheless, the various examples described in this review highlight the importance of alternative splicing events in both physiological and pathological functions of oncogenic kinases. In some cases, the alternative isoforms are present in normal tissue but are aberrantly expressed in the corresponding tumor. It is sometimes unclear whether changing the proportion of a given set of splice variants may contribute to cancer biology, independent of gene expression levels. In other cases, pathogenic alternative splicing is specific to the tumor and can be a consequence or a cancer-causing event [103, 104]. However, one cannot rule out the possibility that most of the so-called aberrant splicing events described in tumors actually reflect the exploitation of yet unidentified regulatory mechanisms normally used at specific moments of the development or in response to external stimuli. Thus, beside gene mutations, hijacking alternative splicing mechanisms in kinase-encoding genes represents another way for cancer cells to progress toward malignancy.

It becomes evident that recently available large-scale analyses will prove useful in identifying new mechanisms of carcinogenesis involving alternative splicing of oncogenic kinases. Although not focused on protein kinases, such approaches have been already applied for the identification of alternative splicing markers in various types of cancers [105–110]. A recent screening that used small interfering RNA for isoform-specific silencing, identified alternative splicing in genes associated with cell survival as important targets for breast and ovarian cancer [111]. For example, changing the splicing pattern of the tyrosine kinase SYK altered cell survival and mitotic progression whereas global knockdown of the same gene had no effect, suggesting a specific role for SYK splice variants. With regard to physiological functions of splice variants of oncogenic kinases in nonpathological conditions, the examples reviewed here clearly highlight the power of mouse genetics. Further development of knockout models restricted to alternatively spliced exons will certainly be beneficial in identifying such functions.

## Figures and Tables

**Table 1:** Regulatory mechanisms of oncogenic kinases activity by alternative splicing.

	Kinase name	Kinase type	Splicing	Regulation type
	Fyn	Cytosolic tyrosine kinase	Alternative use of exon 7a or 7b upstream of the kinase domain	Kinase activity modulation by interfering with autoinhibition
	Fak	Focal adhesion tyrosine kinase	Multiple alternative splicing upstream of the kinase domain	Kinase activity modulation by interfering with autophosphorylation
	B-Raf	Cytosolic serine/threonine kinase	Alternatively spliced exon 8b and 9b upstream of the kinase domain	Kinase activity modulation by interfering with phosphorylation and autoinhibition
Intact kinase domain	Ret	Membrane-bound tyrosine kinase receptor	C-terminal alternative splicing generating three isoforms	Modulation of signaling partners binding
	ErbB4	Membrane-bound tyrosine kinase receptor	N- and C-terminal alternative splicing generating four isoforms	Modulation of partners binding, cleavage, and subcellular localization
	FGFR1 FGFR2 FGFR3	Membrane-bound tyrosine kinase receptors	Alternative use of exon 8 or 9 generating distinct extracellular immunoglobulin-like domain III	Modified FGF binding specificity

	A-Raf	Cytosolic serine/threonine kinase	Intronic sequences retention introducing stop codons	Dominant negative
Kinase domain truncation	TrkB TrkC	Membrane-bound tyrosine kinase receptors	C-terminal alternative splicing replacing kinase domain by short amino acid sequences	Ligand sequestering, dominant negative and/or specific signaling functions
	VEGFR1 VEGFR2	Membrane-bound tyrosine kinase receptors	C-terminal alternative splicing eliminating the kinase and transmembrane domains	Synthesis of secreted/soluble extracellular ligand-binding domains
